# Hair cell damage recruited Lgr5-expressing cells are hair cell progenitors in neonatal mouse utricle

**DOI:** 10.3389/fncel.2015.00113

**Published:** 2015-04-01

**Authors:** Jinchao Lin, Xiaodong Zhang, Fengfang Wu, Weinian Lin

**Affiliations:** Department of Otolaryngology-Head and Neck Surgery, Quanzhou First Hospital Affiliated to Fujian Medical UniversityQuanzhou, Fujian, China

**Keywords:** hair cell, Lgr5, progenitor cells, regeneration, utricle

## Abstract

Damage-activated stem/progenitor cells play important roles in regenerating lost cells and in tissue repair. Previous studies reported that the mouse utricle has limited hair cell regeneration ability after hair cell ablation. However, the potential progenitor cell population regenerating new hair cells remains undiscovered. In this study, we first found that Lgr5, a Wnt target gene that is not usually expressed in the neonatal mouse utricle, can be activated by 24 h neomycin treatment in a sub-population of supporting cells in the striolar region of the neonatal mouse utricle. Lineage tracing demonstrated that these Lgr5-positive supporting cells could regenerate new hair cells in explant culture. We isolated the damage-activated Lgr5-positive cells with flow cytometry and found that these Lgr5-positive supporting cells could regenerate hair cells *in vitro*, and self-renew to form spheres, which maintained the capacity to differentiate into hair cells over seven generations of passages. Our results suggest that damage-activated Lgr5-positive supporting cells act as hair cell progenitors in the neonatal mouse utricle, which may help to uncover a potential route to regenerate hair cell in mammals.

## Introduction

Hair cells are the mechanosensory cells in the inner ear that are responsible for hearing, balance, and body orientation. The utricle, which is a vestibular organ, requires these mechanosensory hair cells to detect linear acceleration. The mammalian cochlea also requires these sensory hair cells to detect auditory information. Previous studies showed that in birds, both the basilar papilla and the utricle have the ability to regenerate lost hair cells (Corwin and Cotanche, 1988; Jørgensen and Mathiesen, 1988; Ryals and Rubel, 1988; Roberson et al., 1992; Weisleder and Rubel, 1993). However, the mature mammalian cochlea lacks the ability to regenerate new hair cells, and only the neonatal mammalian cochlea has a very limited capacity to self-renew hair cells (Chai et al., 2012; Shi et al., 2012, 2013; Cox et al., 2014). In contrast, the mature mammalian utricle has the ability to replace a portion of lost hair cells through regeneration. Recently, multiple studies reported that spontaneous hair cell regeneration occurs in the mouse utricle after hair cell damage and that hair cell numbers continue to increase after birth (Forge et al., 1993; Warchol et al., 1993; Rubel et al., 1995; Lambert et al., 1997; Oesterle et al., 2003; Kawamoto et al., 2009; Lin et al., 2011; Burns et al., 2012a; Golub et al., 2012). Utricle supporting cells have been demonstrated as a reliable source to regenerate hair cells via both direct differentiation and mitotic division (Lin et al., 2011; Burns et al., 2012a,b; Golub et al., 2012). However, identity of the sub-population of supporting cells contributing to the newly regenerated hair cells remains unclear. In the mouse utricle, the striolar region mainly contains the type I hair cells and the extra-striolar region mainly contains the type II hair cells (Dechesne and Thomasset, 1988; Dechesne et al., 1988; Desai et al., 2005), however, the difference of the supporting cells in these two regions remains largely unknown.

In rapid proliferating tissues like the integumentary and intestinal systems, adult stem cells are capable of self-renewing. Following tissue injury, these endogenous stem cells are activated to initiate the self-repair system and to regenerate lost cells (Tumbar et al., 2004; Barker et al., 2007; Jaks et al., 2008; Lim et al., 2013). In organs with low levels of proliferation, such as the liver and pancreas, there are no adult stem cells in the absence of tissue damage. However upon tissue injury, the damage recruited Lgr5-positive stem cells are activated to regenerate the lost cells (Huch et al., 2013a,b). Similar to the liver and pancreas, the utricle is also an organ with little proliferation and limited regeneration ability after damage. Previous studies have reported that after damage, the spontaneously regenerated hair cells come from supporting cells (Li et al., 2003; Oshima et al., 2007; Lin et al., 2011; Burns et al., 2012a; Golub et al., 2012), however, the source of the progenitor cell population responsible for regenerating hair cells in the mammalian utricle has not been identified.

Previous studies reported that Wnt signaling plays important roles in tissue repair (Logan and Nusse, 2004). Lgr5 is a Wnt downstream target gene that acts as a stem cell marker in rapidly proliferating organs, such as integumentary tissue and intestine (Barker et al., 2007; Jaks et al., 2008), as well as in organs with little proliferation, such as the liver and pancreas, (Huch et al., 2013a,b). In the cochlea, recent studies showed that Lgr5 is expressed in a subpopulation of supporting cells, including the third Deiters’ cells, inner pillar cells, inner phalangeal cells and greater epithelial ridge (GER) cells. Lgr5-expressing cells in the cochlea can regenerate hair cells both *in vivo* and *in vitro*, thus acting as a hair cell progenitor in the mouse cochlea (Chai et al., 2011, 2012; Shi et al., 2012, 2013; Bramhall et al., 2014). In neonatal cochlea, these Lgr5-expressing progenitor cells also have the ability to regenerate new hair cells via either direct differentiation or mitotic division after hair cell loss (Cox et al., 2014). However, the roles of Lgr5 and Lgr5-expressing cells after damage in the utricle remain unknown.

In this study, we report the activation of Lgr5 expression in a subset of supporting cells in the striolar region of neonatal utricle after neomycin induced hair cell loss, and use linage tracing to demonstrate that these Lgr5-positive cells can regenerate new hair cells. Furthermore, we found that when isolated by flow cytometry, these Lgr5-positive cells could regenerate hair cells *in vitro*, and self-renew to form spheres. Together, we concluded that the damage activated Lgr5-expressing cells are hair cell progenitors in the neonatal mouse utricle.

## Materials and Methods

### Animals

We used Lgr5-EGFP-CreERT2 (Jackson Laboratory, Stock 008875) mice to detect Lgr5 expression, and Lgr5-EGFP-CreERT2 and ROSA26R-tdTomato (Jackson Laboratory, Stock 007908) mice to trace the lineage of Lgr5-expressing progenitor cells. Mice were housed with open access to food and water. For culture experiments, mice were killed by CO_2_ inhalation followed by decapitation. This study was carried out in strict accordance with the recommendations in the Guide for the Care and Use of Laboratory Animals of the National Institutes of Health. The protocol was approved by the Committee on the Ethics of Animal Experiments of Quanzhou First Hospital Affiliated to Fujian Medical University. The IACUC committee members at Quanzhou First Hospital Affiliated to Fujian Medical University approved this study. All surgery was performed under sodium pentobarbital anesthesia, and all efforts were made to minimize suffering.

### Whole Organ Cultures

Temporal bones were isolated from P1 mice and placed in ice cold HBSS (Sigma). The otoconia and the otoconial membranes were removed, utricles were isolated using Dumont fine forceps and cultured free-floating in a 96-well plate containing 100 µl of DMEM/F12 (Invitrogen) with 1% N2 (Invitrogen), 2% B27 (Invitrogen), bFGF (1 ng/ml; Sigma), IGF-1 (50 ng/ml; Sigma), EGF (20 ng/ml; Sigma), heparin sulfate (50 ng/ml; Sigma), and ampicillin (50 ng/ml; Sigma) at 37°C with 95% air/5% CO_2_. Each well contained one utricle. To damage hair cells, 1mM neomycin (Sigma) was added to the culture medium for 24 h. To activate the Cre for lineage tracing, 4OH-tamoxifen (800 nM, Sigma) was added to the culture medium. The culture medium was changed daily. For all whole organ culture experiments, we only used one utricle per mouse; n values represent the number of animals. For each experiment, at least 3 mice were used.

### Genotyping and qRT-PCR

Transgenic mice were genotyped using genomic DNA from tail tips by adding 100 µl 50 mM NaOH, incubated at 98°C for 20–40 min, followed by the addition of 10 µl 1 M HCl. The genotyping primers were as follows: Lgr5: wild-type (F) CTGCTCTCTGCTCCCAGTCT; (R) ATACCCCATCCCTTTTGAGC; mutant (R) GAACTTCAGGGTCAGCTTGC; td-Tomato: wild-type (F) AAGGGAGCTGCAGTGGAGT; (R) CCGAAAATCTGTGGGAAGTC; mutant (F) GGCATTAAAGCAGCGTATC; (R) CTGTTCCTGTACGGCATGG.

For quantitative reverse transcriptase polymerase chain reaction (qRT-PCR), total RNA was extracted from isolated utricles using TRIzol (Invitrogen), followed by cDNA synthesis using the GoScript™ Reverse Transcription System (Promega, A5001). qRT-PCR reactions were performed with GoTaq® qPCR Master Mix (Promega, A6001) on a 7500HT Fast Real-Time PCR System (Applied Biosystems). Each qPCR reaction was carried out in triplicate and the relative quantification of gene expression analyzed using the ^ΔΔ^C*_T_* method with the housekeeping gene β-actin as the endogenous reference.

Primer pairs were designed using Primer3 software. Lgr5 (F) CCTACTCGAAGACTTACCCAGT; (R) GCATTGGGGTGAATGATAGCA-3; Sox2 (F) GCGGAGTGGAAACTTTTGTCC; (R) CGGGAAGCGTGTACTTATCCTT; Brn3.1 (F) CGACGCCACCTACCATACC; (R) CCCTGATGTACCGCGTGAT-3′ Jagged1 (F) TCAAACGTGAGAGTGTCTAACG; (R) CCGGGCCGAAGAGATTTCTG; β-actin (F) GGCTGTATTCCCCTCCATCG; (R) CCAGTTGGTAACAATGCCATGT.

### Cell Counting

**Figure 1:**
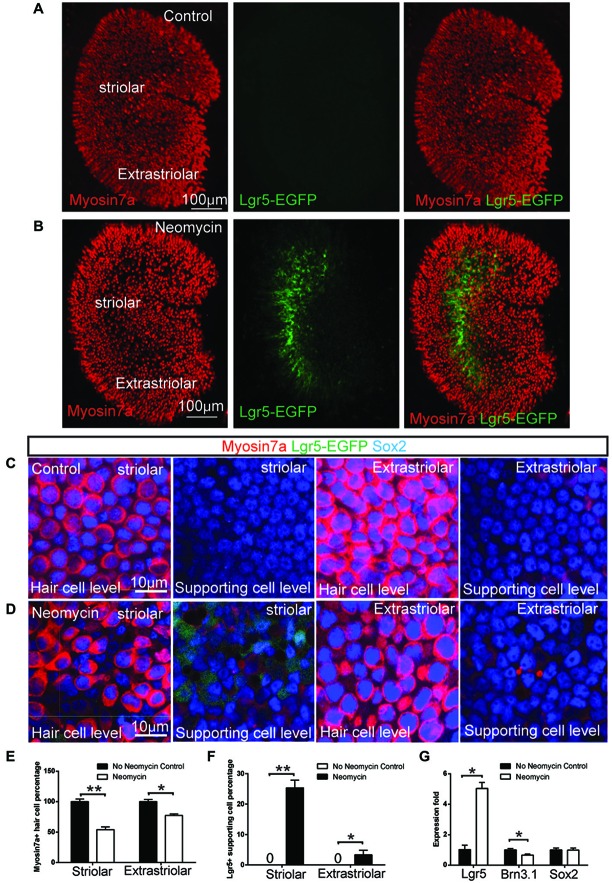
**Neomycin-induced hair cell damage activated Lgr5 expression in mouse utricles. (A)** In Lgr5-EGFP-CreERT2 control utricles without damage, no Lgr5-EGFP expression was detected at P1.** (B)** In contrast, in Lgr5-EGFP-CreERT2 utricles with neomycin damage, many Lgr5-EGFP-positive supporting cells were detected in the striolar region. **(C)** High magnification picture showed there was no Lgr5-EGFP expression in both striolar and extra-striolar region in control utricle without damage. **(D)** In Lgr5-EGFP-CreERT2 utricle withs neomycin damage, Lgr5-EGFP was mainly expressed in a subset of supporting cells in the striolar region. **(E)** Quantification and comparison of Myosin7a-positive hair cell in the striolar and extra-striolar region of utricles with or without neomycin damage. **(F)** Quantification and comparison of Lgr5-EGFP-positive supporting cell in the striolar and extra-striolar region of utricles with or without neomycin damage. **(G)** Quantitative PCR showed that neomycin treatment significantly increased the expression level of Lgr5 and slightly decreased the expression level of the hair cell marker Brn3.1 as compared to control utricles. *p < 0.05, **p < 0.01, *n* = 3 mice in** (E–G)**. Scale Bars: **(A,B)**: 100 µm; **(C,D)**: 10 µm.

**Figure 2:**
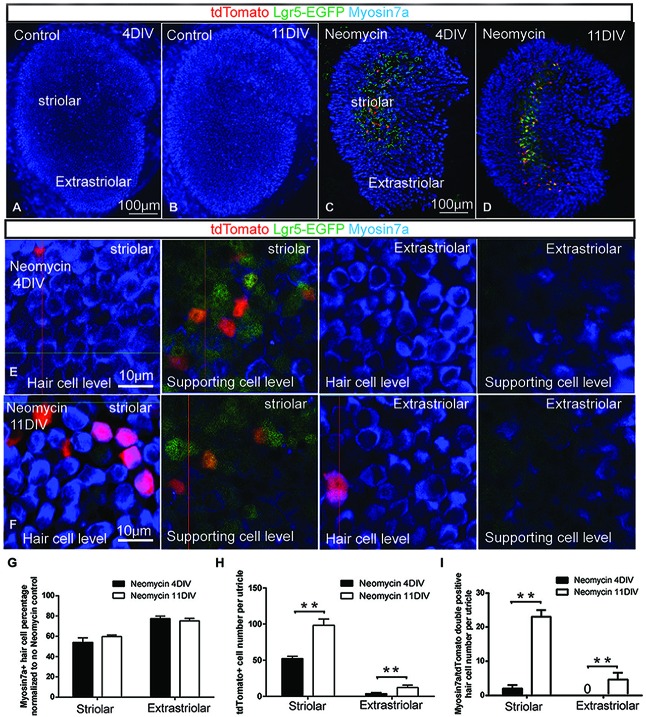
**Damage-activated Lgr5-positive cells generated hair cells in whole organ culture. (A–B)** In Lgr5-EGFP-CreERT2 control utricles, there was no Lgr5-GFP expression and no tdTomato reporter expression after 4 or 11 days in culture. **(C)** In Lgr5-EGFP-CreERT2 utricles with neomycin damage, tdTomato reporter expression was detected mostly in the supporting cells in the striolar region at 4 days in culture. **(D)** At 11 days in culture, the total number of tdTomato-positive cells was increased and tdTomato reporter expression was also detected in Myo7a-positive hair cells. **(E)** High magnification picture showed most of the tdTomato-positive cells were supporting cells in the striolar region at 4 days in culture. **(F)** High magnification picture showed significant numbers of tdTomato-positive cells were hair cells in the striolar region at 11 days in culture. **(G)** The total hair cell number was not significantly increased from 4 days to 11 days in culture. **(H)** The total tdTomato-positive cell number was significantly increased from 4 to 11 days in culture.** (I)** The myosin7a and tdTomato double positive hair cells number was significantly increased from 4 to 11 days in culture. **p < 0.01, *n* = 3 mice in **(G–I)**. Scale Bars: **(A–D)**: 100 µm; **(E,F)**: 10 µm.

For whole organ culture experiments, we randomly took 2 representative pictures from the striolar region or extra-striolar regions for analyses. When we took the pictures, Lgr5-EGFP and tdTomato expression was used as a reference to define the striolar region. For cell counting, we either counted the number of hair cells in representative pictures and normalized to undamaged control to get the hair cell percentage (for example, Figures 1E, 2G); or counted Lgr5+ supporting cell number in representative pictures and normalized to total Sox2+ supporting cells to get the Lgr5+ supporting cell percentage (for example, Figure 1F); or counted the total tdTomato+ or myosin7a/tdTomato double positive cell number per utricle (for example, Figures 2H,I). For all experiments, n values represent the number of mice.

### Isolation of Lgr5-Expressing Cells by Flow Cytometry

20–30 utricles from Lgr5-EGFP-CreERT2 mice were cultured with 1 mM Neomycin for 24 h and recovered for 24 h and then trypsinized at 37°C for 10 min and mechanically dissociated in PBS with 2% fetal bovine serum (FBS, Invitrogen), DNAse (10 units/ml, Qiagen) and EDTA (2 mM, Sigma). The cells were filtered through a cell strainer (40 µm diameter) prior to sorting. The dissociated cells were sorted on a BD FACS AriaIII (BD Biosciences) using the channel for GFP, and positive fractions were collected.

### Culture of Sorted Cells

Florescence Activated Cell Sorting (FACS) isolated Lgr5-expressing cells (20 cells/ul, 2000 cells per well) were plated on a laminin-coated dish and cultured for 10 d in DMEM/F12 with 2% B27, 1% N2, EGF, bFGF, IGF-1 and heparan sulfate (same as whole organ culture). Cells that had not attached were removed 1 d after plating. To label dividing cells, EdU (1.0 µM; Invitrogen) was added to the culture medium. The cells were fixed and stained for EDU and hair cell marker Myosin7a after 10 d of culture.

### Sphere Formation and Differentiation

Two hundred FACS-isolated Lgr5-expressing cells were cultured to form spheres in 96 well ultra-low attachment plates (Costar) with a density of 2 cells/µl for 5 d in DMEM/F12 medium (Invitrogen) with 1% N2 and 2% B27, EGF, bFGF, IGF-1 and heparin sulfate. The spheres were collected every 5 d, mechanically dissociated with a 25 G needle (BD Labware) and re-seeded in fresh medium. For differentiation, spheres were plated on a laminin-coated dish and cultured for 5 d in DMEM/F12.

### Histological Methods

Proteins were detected in whole-mount utricles using standard immunofluorescence labeling methods. Utricles were fixed with 4% PFA (Sigma) for 1 h at room temperature, rinsed with PBS for 3 times, and incubated for 1 h in blocking solution (2% bovine serum albumin, 5% normal goat serum, 0.5% Triton X-100). Utricles were incubated overnight at 4°C with one of the following primary antibodies diluted 1/100–1/1000 in blocking solution: rabbit anti-myosin VIIa (1:1000, Proteus Biosciences), Sox2 (1:400; Santa Cruz Biotechnology). Secondary antibodies, conjugated to Alexa Fluor 488, 594, or 647 and diluted 1/500, were purchased from Invitrogen. To label cell nuclei, organs were soaked in 4′, 6-diamidino-2-phenylindole (DAPI) (Sigma) at 1 µg/ml for 10 min. Images were acquired using confocal microscopy (Zeiss LSM710) and analyzed with Image J (NIH) and Photoshop CS4 (Adobe Systems).

### Statistical Analyses

Throughout the text and in graphs, data are expressed as mean ± SD. The immunohistochemical data was analyzed using Student’s *t*-test when comparing two groups or by one-way ANOVA followed by Dunnett’s Multiple Comparisons Test when comparing more than two groups. Values of *p* < 0.05 were considered statistically significant.

## Results

### Activation of Lgr5 Expression in a Subset of Supporting Cells in the Striolar Region After Hair Cell Damage

Lgr5 has been reported as a stem cell marker in a variety of organs, including intestine, liver, pancreas and hair follicles (Barker et al., 2007, 2010; Jaks et al., 2008; Huch et al., 2013a,b). In this study, we explored the expression of Lgr5 using the Lgr5-EGFP-CreERT2 mice, where GFP is under the control of the Lgr5 locus. In the mouse utricle, similar to the liver (Huch et al., 2013b), we found Lgr5 expression is not detectable at P1 plus 1 day *in vitro* (DIV; Figures 1A,C). However, when we cultured the P1 utricle from Lgr5-EGFP-CreERT2 mice and treated it with 1 mM neomycin for 24 h, we found the treatment significantly damaged the hair cell both in striolar and extra-striolar region (Figure 1E), and Lgr5 expression was robustly activated specifically in a subset of supporting cells in the striolar region (Figures 1B,D). Quantification data suggested 25.2 ± 7.3% of striolar supporting cells were Lgr5-GFP-positive (*n* = 3), and no hair cells were observed to be GFP-positive (*n* = 3) (Figure 1F). qPCR experiments confirmed that the expression of Lgr5 is undetectable at P1 in the absence of neomycin, and has been robustly activated following neomycin induced hair cell damage (*n* = 3) (Figure 1G). Interestingly, we found that during embryonic development, Lgr5 was also mainly expressed in a subset of supporting cells in the striolar region (Sup-Figures 1A–D). This result suggested that during development the expression of Lgr5 was rapidly down-regulated and became undetectable after birth. Compared to P1 Lgr5 expression after damage, the Lgr5 expression at E17.5 was restricted to the striolar region and mainly in the supporting cells; however at E17.5 there was also very low expression of Lgr5 in some of the hair cells, while in P1 utricle after damage, there was no Lgr5 expression in hair cells.

### Damage Activated Lgr5-Positive Cells Generated Hair Cells in Whole Organ Culture

Previous studies reported that Lgr5-positive cells in the cochlea have the ability to regenerate hair cells (Chai et al., 2012; Shi et al., 2012). To ask whether the damage activated Lgr5-positive supporting cells in the utricle could regenerate hair cells, we crossed Lgr5-EGFP-CreERT2 mice with ROSA26R-tdTomato reporter mice in which a floxed STOP cassette prevents transcription of a downstream red fluorescent protein (tdTomato). Utricles were dissected from P1 double transgenic mice, and treated with 1 mM neomycin for 24 h (1 DIV) to damage the hair cells. In control samples without neomycin treatment, there was neither Lgr5-GFP expression nor tdTomato reporter expression (*n* = 3) (Figures 2A,B). After neomycin treatment, 800 nM 4OH-tamoxifen was administrated for 72 h to activate the Cre for lineage tracing the Lgr5-positive cells (4 DIV). When utricle tissues were examined at 4 DIV, tdTomato reporter expression was almost only observed in a subset of supporting cells in the striolar region, and very few hair cells were found to be tdTomato positive (*n* = 3) (Figures 2C,E,I). When the lineage tracing was extended to 11 DIV, we found the total number of tdTomato-positive cells was significantly increased (*n* = 3) (Figure 2H), suggesting these lineage traced Lgr5-positive cells were highly proliferative. We also observed significant number of hair cell marker myosin7a and tdTomato double positive hair cells (*n* = 3) (Figures 2D,F,I), indicating that these new regenerated hair cells were indeed derived from Lgr5-positive supporting cells. However, the total hair cell number in the striolar region only slightly increased and was not statistically significant (*P* = 0.13, *n* = 3) (Figure 2G). Of the GFP-positive supporting cells, only a small population has been lineage traced with tdTomato, presumably due to the low efficiency of 4OH-tamoxifen in culture and the incomplete labeling of tdTomato reporters by Lgr5-EGFP-CreERT2 mouse (Barker et al., 2007).

### FACS Isolated Lgr5-Positive Cell Divided and Generated Hair Cells in Cell Culture

**Figure 3:**
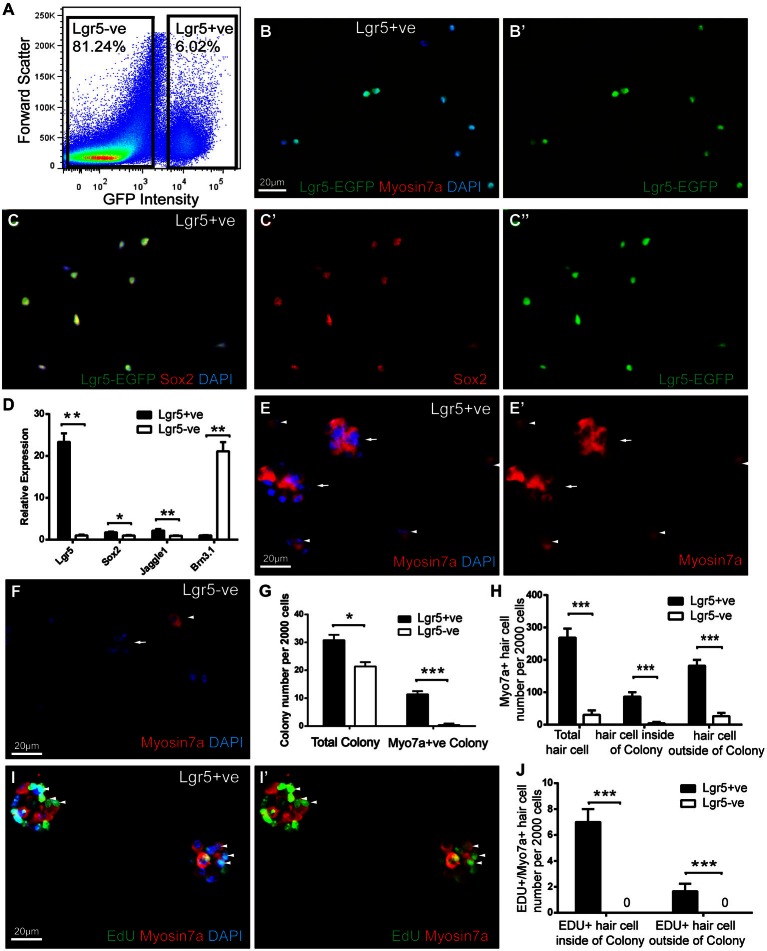
**FACS isolated Lgr5-positive cell divided and generated hair cells in culture. (A)** Lgr5-GFP-positive and Lgr5-GFP-negative cells were isolated using FACS. **(B,C)** Immunostaining showed that among the FACS isolated Lgr5-EGFP-positive cells, 96.6% were GFP-positive, 96.7% were Sox2-positive, and none were Myo7a-positive. **(D)** Quantitative PCR showed that FACS isolated Lgr5-EGFP-positive cells had significantly higher Lgr5 expression, slightly higher Sox2 and Jagged1 expression, and significantly lower Brn3.1 expression compared to the Lgr5-EGFP-negative cells. **(E,F)** FACS isolated Lgr5-EGFP-positive cells generated significantly more colonies and Myo7a-positive cells than Lgr5-EGFP-negative cells.** (G)** Quantification of colony number generated from Lgr5-EGFP-positive and Lgr5-EGFP-negative cells. **(H)** Quantification of Myo7a-positive hair cells generated from Lgr5-EGFP-positive and Lgr5-EGFP-negative cells. **(I)** FACS isolated Lgr5-EGFP-positive cells mitotically generated hair cells in cell culture. **(J)** Quantification of EDU/Myo7a double positive hair cell number generated from Lgr5-EGFP-positive and Lgr5-EGFP-negative cells. *p < 0.05, **p < 0.01, ***p < 0.001, *n* = 3 in **(D**,**G,H**,**J)**. Scale Bars: 20 µm.

Next we tested whether the damage-activated Lgr5-expressing supporting cells could act as hair cell progenitors to divide and differentiate into hair cells, when isolated by FACS. In this experiment, we first cultured the P1 utricles from Lgr5-EGFP-CreERT2 mice with 1 mM neomycin for 24 h, then allowed recovery for 24 h, followed by purifying the GFP-positive cells by FACS. We found that 6.02% of the cells were GFP-positive (*n* = 3) (Figure 3A). 96.6 ± 5.1% of Lgr5-positive supporting cells sorted by flow cytometry were also GFP positive (*n* = 3), and isolated Lgr5-positive supporting cells showed no staining of the hair cell marker myosin7a (0.0 ± 0.0%, *n* = 3) (Figure 3B). However, almost all of the Lgr5-positive cells displayed the supporting cell marker Sox2 (96.7 ± 4.8%, *n* = 3) (Figure 3C). qPCR data also demonstrated that the GFP-positive cells had significantly higher Lgr5 expression level, as well as expression of the supporting cell markers Jagged1 and Sox2, and significantly lower expression of the hair cell marker Brn3.1, compared with the GFP-negative cells (*n* = 3) (Figure 3D).

Next, we cultured the damage activated Lgr5-positive cells with a density of 20 cells/µl for 10 days and found that these damage-activated Lgr5-positive supporting cells formed colonies (more than 5 cells) that expressed myosin7a (Figure 3E). After 10 days in culture, 2000 Lgr5-positive supporting cells generated 31.6 ± 2.8 colonies, among which 38.5 ± 3.9% colonies contained myosin7a-positive hair cells (arrow). These colonies contained a total number of 87.1 ± 15.7 myosin7a-positive hair cells, indicating that the hair cells resided in the colonies might be generated via mitotic regeneration (arrow) (*n* = 3) (Figures 3E,G). Moreover, 2000 Lgr5-positive supporting cells also generated around 182.5 ± 34.2 myosin7a-positive hair cells (arrow head) outside of the colonies, indicating these hair cells resided separately might be generated via direct differentiation (arrow head) (*n* = 3) (Figures 3E,H). In contrast, 2000 Lgr5-negative cells generated 20.2 ± 3.9 colonies, with only 1.2 ± 1.7 colonies containing myosin7a-positive hair cells. Only 4.7 ± 4.2 myosin7a-positive hair cells were generated within colonies, and 26.9 ± 13.5 myosin7a-positive hair cells resided outside the colonies (Figures 3F–H).

We next added 1.0 µM EdU to the culture medium from day 3 to day 5 to label dividing cells, and found evidence of proliferation in damage-activated Lgr5-positive supporting cells (Figure 3I). 8.2 ± 1.9% and 1.2 ± 0.9% of the myosin7a-positive hair cells were co-stained for EdU inside and outside of the colonies, respectively (Figures 3I,J). In contrast, Lgr5-negative cells also proliferated as indicated by EdU incorporation but no myosin7a/EdU double positive hair cells were observed (Figure 3J).

### FACS Isolated Lgr5-Positive Cell Self-Renew to Form Spheres and Could be Passaged for 7 Generations

**Figure 4:**
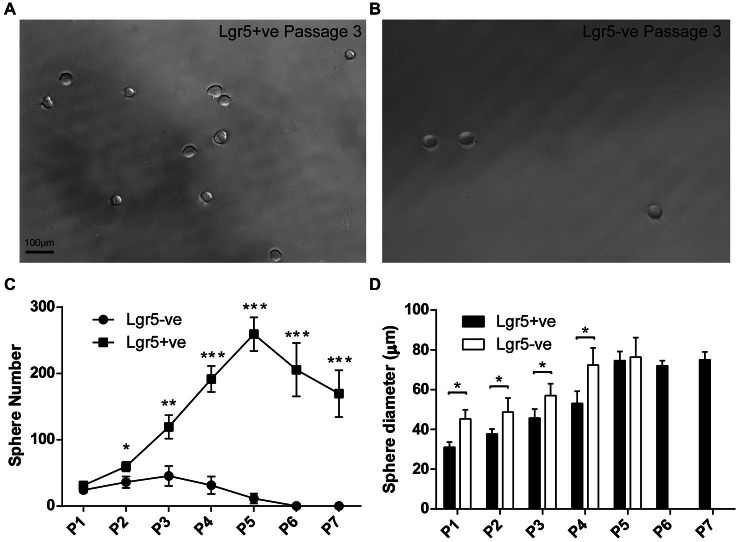
**FACS isolated Lgr5-positive cells self-renew to form spheres. (A,B)** FACS isolated Lgr5-EGFP-positive cells generated significantly more spheres than Lgr5-EGFP-negative cells. **(C)** Quantification of sphere number generated from FACS isolated Lgr5-EGFP-positive and Lgr5-EGFP-negative cells over seven generations.** (D)** Quantification of sphere size generated from FACS isolated Lgr5-EGFP-positive and Lgr5-EGFP-negative cells over seven generations. *p < 0.05, **p < 0.01, ***p < 0.001, *n* = 3 in **(C,D)**. Scale Bars: 100 µm.

To assess the potential of damage-activated Lgr5-positive supporting cells to self-renew and expand the colony, we used a sphere assay to examine Lgr5-positive cells isolated from cultured utricles. As described above, we first sorted the damage activated Lgr5-positive supporting cells with FACS, then cultured 200 isolated cells to form spheres with a density of 2 cells/µl in ultra-low attachment plates. In the first generation, the sphere number derived from damage-activated Lgr5-positive cells was only slightly higher than the Lgr5-negative cells. However, from the second to the fifth generation, the Lgr5-positive cells formed significantly more spheres than the Lgr5-negative cells (Figures 4A–C). Multiple passages suggested that spheres derived from Lgr5-positive cells expanded significantly more rapidly than from Lgr5-negative cells (Figure 4C). From the sixth generation on, there were no spheres formed in the Lgr5-negative group and the total number of spheres started to decrease in the Lgr5-positive group, therefore we only passaged the spheres to the seventh generation. When we measured the size of the spheres, we found Lgr5-positive cells formed smaller and more homogenous spheres than Lgr5-negative cells in the first four generations (Figure 4D). When we compared multiple generations, we found the size of spheres also increased with the passage number (Figure 4D).

### Spheres Derived from Lgr5-Positive Cells Maintained the Capacity to Differentiate into Hair Cells

**Figure 5:**
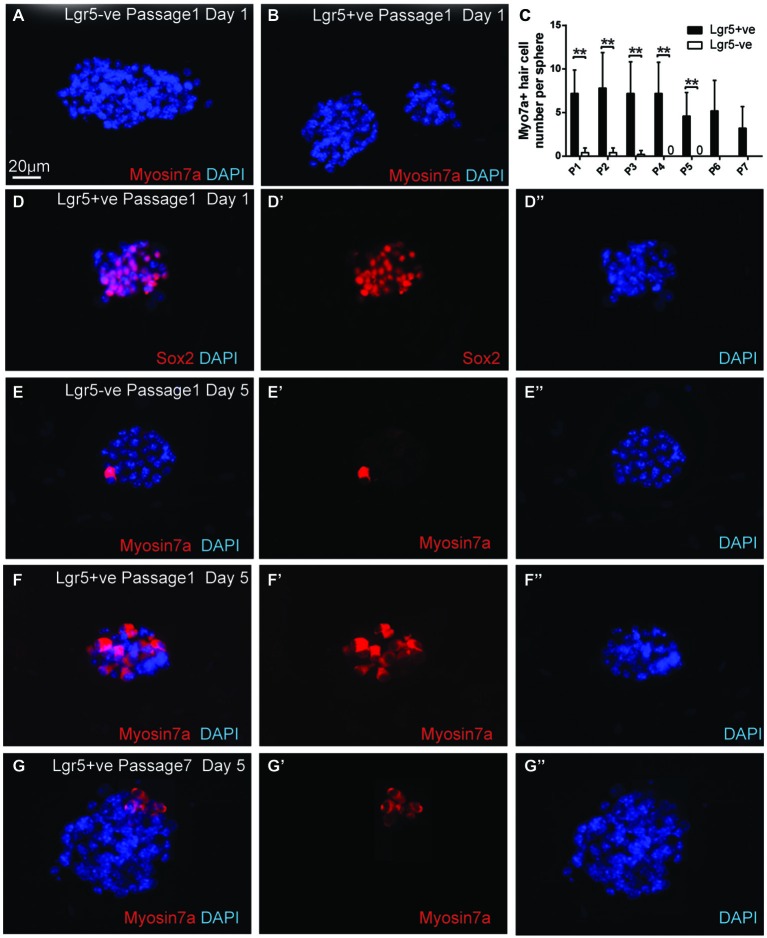
**Spheres derived from Lgr5-positive cells maintained the capacity to differentiate into hair cells. (A,B)** The spheres derived from both Lgr5-EGFP-positive and Lgr5-EGFP-negative cells contained no Myo7a-positive hair cells at the first day of differentiation** (C)** Quantification of Myo7a-positive hair cell number per sphere generated from Lgr5-EGFP-positive and Lgr5-EGFP-negative cells over seven generations. **(D)** At the first day of differentiation, the majority of the spheres were Sox2-positive. **(E,F)** The sphere derived from Lgr5-EGFP-positive cells generated significantly more Myo7a-positive hair cells than the one derived from Lgr5-EGFP-negative cells.** (G)** The sphere derived from Lgr5-EGFP-positive cells maintained the capacity to generate Myo7a-positive hair cells after seven passages. **p < 0.01, *n* = 3 in **(C)**. Scale Bars: 20 µm.

To determine if the passaged spheres derived from damage activated Lgr5-positive supporting cells still maintained the ability to differentiate into hair cells, we collected the spheres from different generations and differentiated the spheres for 5 days. For the first generation of spheres, we found that on the first day of the differentiation the spheres from both damage-activated Lgr5-positive cells and Lgr5-negative cells contained no myosin7a-positive hair cell (Figures 5A,B), but almost all the spheres were positive for the supporting cell marker Sox2 (Figure 5D). Upon 5 days of differentiation, the spheres derived from Lgr5-positive cells generated significantly more myosin7a-positive hair cells than the Lgr5-negative cells (6.7 ± 3.5 vs. 0.4 ± 0.5 myosin7a-positive hair cells in Lgr5-positive and Lgr5-negative cells, respectively) (Figures 5C,E,F).

After multiple generations of passages, we found that from the first to the fourth generation there was no significant difference in the number of differentiated hair cells per sphere (Figure 5C), suggesting that hair cell regeneration ability remained robust in the early sphere generations. Although the number of differentiated hair cells per sphere decreased from the fifth to the seventh generations, the seventh generation of spheres derived from damage-activated Lgr5-positive supporting cells still generated 3.2 ± 2.6 hair cells per sphere, suggesting that the seventh generation of sphere still maintained the capacity to differentiate into hair cells (Figures 5C,G).

## Discussion

In birds, both the basilar papilla and the utricle have been reported to maintain hair cell regeneration capacity (Corwin and Cotanche, 1988; Jørgensen and Mathiesen, 1988; Ryals and Rubel, 1988; Roberson et al., 1992; Weisleder and Rubel, 1993). Supporting cells in the mammalian cochlea and utricle share evolutionarily conserved marker genes and signaling pathways with those supporting cells in birds, which act as hair cell progenitors. In the mature mammalian cochlea, hair cells and supporting cells are quiescent and the mature mammalian cochlea does not regenerate hair cell spontaneously, thus severe hair cell loss leads to permanent hearing loss (Bermingham-McDonogh and Rubel, 2003; Brigande and Heller, 2009). The mammalian vestibular system still maintains a limited ability to regenerate lost hair cells (Forge et al., 1993; Warchol et al., 1993; Rubel et al., 1995; Lambert et al., 1997; Oesterle et al., 2003; Kawamoto et al., 2009; Lin et al., 2011; Burns et al., 2012a; Golub et al., 2012), therefore, the mammalian utricle could serve as a useful model to study hair cell regeneration. Multiple studies reported that utricular supporting cells have the capacity to replenish lost hair cells via both direct differentiation and mitotic division (Lin et al., 2011; Burns et al., 2012a,b; Golub et al., 2012). However, the clearly defined progenitor cell population in the mammalian utricle has not been identified. In this study, we demonstrate that in the mouse utricle, the Lgr5 expression was activated in a specific subset of striolar supporting cells after neomycin-induced hair cell loss. These damaged-activated Lgr5-positive supporting cells exhibit progenitor cell properties and have the ability to regenerate lost hair cells. Previous studies reported that the hair cell types in the striolar and extra-striolar region are different: type I hair cells are mainly located in the striolar region, while type II hair cells are mainly located in the extra-striolar region (Dechesne and Thomasset, 1988; Dechesne et al., 1988; Desai et al., 2005). In 2012, Burns found that in adult utricle, newly arising hair cells are mainly located in the peripheral edge and striola, and after hair cell ablation mitotic regeneration of hair cells was observed in the striola (Burns et al., 2012a,b). In our study, we found the damage-activated Lgr5-positive supporting cells are also restricted to the striolar region, suggesting the striolar and extra-striolar supporting cells are two distinct populations with different properties. Interestingly, the damage-activated Lgr5-positive supporting cells only cover around 25% of the whole supporting cell population in the striolar region, which also indicates that striolar supporting cells do not consist of a single population but may contain several subpopulations with distinct functions. It will be interesting to further investigate the different functions of supporting cell subpopulations in the future. More interestingly, we found that at E17.5, Lgr5 was also mainly expressed in a subset of supporting cells in the striolar region, and this expression pattern is similar to the Lgr5 expression after hair cell damage at P1, which indicates that in the postnatal stages, the damage-activated expression of Lgr5 in striolar supporting cells may be partially a recapitulation of developmental mechanisms. Further study will be performed to investigate the detailed mechanism.

Previous reports showed that after hair cell ablation, utricular supporting cells have the ability to regenerate lost hair cells (Burns et al., 2012a,b; Golub et al., 2012). Compared to Sox2, which is widely expressed in all supporting cells, Lgr5 is limited to a subpopulation of the striolar supporting cells. This suggests that this subset of supporting cells with progenitor cell properties is restricted as in non-mammalian vertebrates, in which a small subset of supporting cell population regenerates lost hair cells by proliferation followed by trans-differentiation (Stone and Cotanche, 2007).

Recent studies showed that after tissue damage, the Wnt signaling pathway is activated as a self-repair system and plays key roles in tissue repair in several organs including the liver and pancreas (Minear et al., 2010; Huch et al., 2013a,b). Lgr5, a downstream target gene of Wnt signaling, was first described as a stem cell marker in the intestine, where Lgr5-expressing cells are precursors for all cells in the intestinal crypts (Barker et al., 2007). Deletion of Lgr5 in the intestine leads to the rapid and complete loss of intestinal crypts (de Lau et al., 2011). By far, Lgr5 has been demonstrated as the stem cell marker in multiple organs, including the stomach, hair follicles, liver, pancreas and cochlea (Barker et al., 2007, 2010; Jaks et al., 2008; Chai et al., 2012; Shi et al., 2012, 2013; Huch et al., 2013a,b). Recently, damage recruited Lgr5-positive cells have also been demonstrated as stem cells that can form organoids containing all mature cell types in regeneration-inactive organs such as the liver and pancreas (Huch et al., 2013a,b). In this study, our data indicated that in the utricle, another regeneration-inactive organ, damage recruited Lgr5-positive cells exhibit progenitor cell properties and are capable of regenerating hair cells.

In the mammalian cochlea and utricle, there is little or no detectable proliferation of postnatal cells in the sensory epithelium. However, when supporting cells were isolated they could self-renew to form spheres with the ability to regenerate hair cells *in vitro* (White et al., 2006; Oshima et al., 2007, 2009; Jeon et al., 2011; Sinkkonen et al., 2011), although it was unclear which cell population could proliferate. A recent study showed that Lgr5-positive cells in the cochlea could self-renew to form spheres, with these spheres giving rise to hair cells derived from Lgr5-positive supporting cells (Shi et al., 2012). In the present study, our results showed that FACS sorted damage-activated Lgr5-positive supporting cell have significantly higher sphere formation capacity compared to Lgr5-negative cells. The spheres derived from Lgr5-positive cells were smaller and more homogenous solid spheres, whereas the spheres derived from Lgr5-negative cells were larger and more hollow. When we collected spheres for differentiation, our data showed that the spheres derived from Lgr5-positive cells generated significantly more hair cells than the spheres derived from Lgr5-negative cells, indicating that damage-activated Lgr5-positive supporting cells were enriched in the population of hair cell progenitors in the utricle. After seven passages, spheres derived from Lgr5-positive cells still maintained the capacity for differentiation into hair cells. However, we cannot rule out the possibility that other progenitor cell population in the utricle could contribute to regenerate hair cells after sphere formation.

In summary, we report here that neomycin-induced hair cell loss activates Lgr5 expression in a subpopulation of striolar supporting cells in the utricle. These damage recruited Lgr5-positive supporting cells act as hair cell progenitors both *ex vivo* and *in vitro*. These Lgr5-positive cells could self-renew to form spheres, which maintained the capacity to differentiate into hair cells over seven generation of passages. Therefore, we demonstrated that damage-recruited Lgr5-positive supporting cells could act as hair cell progenitors in neonatal mouse utricle, and serve as candidate therapeutic targets for hair cell regeneration in mammals.

## Author Contributions

Conceived and designed experiments: JL. Provided reagents: JL. Performed experiments: JL, XZ, FW, WL. Analyzed the data: JL, XZ, FW, WL. Wrote the paper: JL, XZ, FW, WL.

## Conflict of Interest Statement

The authors declare that the research was conducted in the absence of any commercial or financial relationships that could be construed as a potential conflict of interest.
