# Supplementary material for: Hair cell damage recruited Lgr5-expressing cells are hair cell progenitors in neonatal mouse utricle
Source: Front Cell Neurosci. 2015 Apr 1;9:113. doi: 10.3389/fncel.2015.00113 (PMC4381628; doi:10.3389/fncel.2015.00113)
Supplement: Supplementary file 1 [file Data_Sheet_1.DOCX]

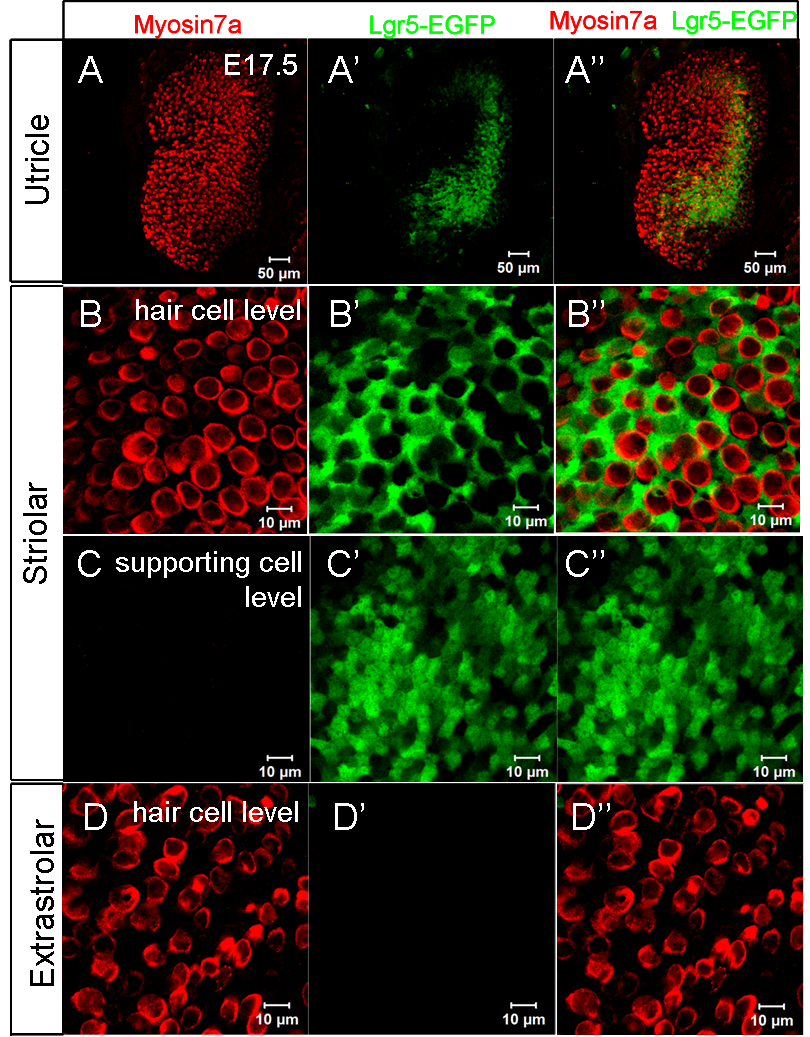


**Supplementary Figure1 Lgr5 expression in the E17.5 utricle**

**(A)** In E17.5 Lgr5-EGFP-CreERT2 utricle, Lgr5-EGFP expression was detected specifically in the striolar region of utricle. **(B-C)** High magnification picture showed that Lgr5-EGFP mainly expressed in a subset of supporting cells in the striolar region at E17.5 utricle. **(D)** Lgr5-EGFP expression was not detected in the extrastriolar region at E17.5.
